# Identification of novel pathogenic MSH2 mutation and new DNA repair genes variants: investigation of a Tunisian Lynch syndrome family with discordant twins

**DOI:** 10.1186/s12967-019-1961-9

**Published:** 2019-06-27

**Authors:** Amira Jaballah-Gabteni, Haifa Tounsi, Maria Kabbage, Yosr Hamdi, Sahar Elouej, Ines Ben Ayed, Mouna Medhioub, Moufida Mahmoudi, Hamza Dallali, Hamza Yaiche, Nadia Ben Jemii, Afifa Maaloul, Najla Mezghani, Sonia Abdelhak, Lamine Hamzaoui, Mousaddak Azzouz, Samir Boubaker

**Affiliations:** 1 Laboratory of Human and Experimental Pathology, Institut Pasteur de Tunis, Tunis, Tunisia; 2 Gastroenterology Department, Mohamed Tahar Maamouri Hospital, 8000 Nabeul, Tunisia; 3 Laboratory of Biomedical Genomics and Oncogenetics, Institut Pasteur de Tunis, Tunis EL Manar University, Tunis, Tunisia; 4 Marseille Medical Genetics, Aix Marseille University, INSERM, Marseille, France

**Keywords:** CRC, Lynch syndrome I, MMR genes, DRGs, Tunisian family

## Abstract

**Background:**

Lynch syndrome (LS) is a highly penetrant inherited cancer predisposition syndrome, characterized by autosomal dominant inheritance and germline mutations in DNA mismatch repair genes. Despite several genetic variations that have been identified in various populations, the penetrance is highly variable and the reasons for this have not been fully elucidated. This study investigates whether, besides pathogenic mutations, environment and low penetrance genetic risk factors may result in phenotype modification in a Tunisian LS family.

**Patients and methods:**

A Tunisian family with strong colorectal cancer (CRC) history that fulfill the Amsterdam I criteria for the diagnosis of Lynch syndrome was proposed for oncogenetic counseling. The index case was a man, diagnosed at the age of 33 years with CRC. He has a monozygotic twin diagnosed at the age of 35 years with crohn disease. Forty-seven years-old was the onset age of his paternal uncle withCRC. An immunohistochemical (IHC) labeling for the four proteins (MLH1, MSH2, MSH6 and PMS2) of the MisMatchRepair (MMR) system was performed for the index case. A targeted sequencing of *MSH2*, *MLH1* and a panel of 85 DNA repair genes was performed for the index case and for his unaffected father.

**Results:**

The IHC results showed a loss of MSH2 but not MLH1, MSH6 and PMS2 proteins expression. Genomic DNA screening, by targeted DNA repair genes sequencing, revealed an *MSH2* pathogenic mutation (c.1552C>T; p.Q518X), confirmed by Sanger sequencing. This mutation was suspected to be a causal mutation associated to the loss of MSH2 expression and it was found in first and second degree relatives. The index case has smoking and alcohol consumption habits. Moreover, he harbors extensive genetic variations in other DNA-repair genes not shared with his unaffected father.

**Conclusion:**

In our investigated Tunisian family, we confirmed the LS by IHC, molecular and in silico investigations. We identified a novel pathogenic mutation described for the first time in Tunisia. These results come enriching the previously reported pathogenic mutations in LS families. Our study brings new arguments to the interpretation of MMR expression pattern and highlights new risk modifiers genes eventually implicated in CRC. Twins discordance reported in this work underscore that disease penetrance could be influenced by both genetic background and environmental factors.

**Electronic supplementary material:**

The online version of this article (10.1186/s12967-019-1961-9) contains supplementary material, which is available to authorized users.

## Background

The surge in CRC incidence in young adults is particularly alarming. Among early onset-CRC, approximately 30% of patients are affected by tumors harboring mutations causing hereditary cancer predisposing syndromes, and 20% have familial CRC [1].

Lynch syndrome (LS) is considered as the most common hereditary CRC form [2]. It is an autosomal dominant syndrome subdivided into LS I, or site-specific colonic cancer, and LS II, or extracolonic cancer, with gastric, endometrial, biliary, pancreatic, and urinary tract carcinomas [3].

This syndrome is responsible of 2 to 6% of all CRC. It is known to increase the risk of other cancers in family members. The lifetime estimated risk for cancer ranges from 50 to 80% for CRC and from 40 to 60% for endometrial cancer [4]. Currently, in the context of lack of LS specific clinical symptoms, there is an important need to identify consistent molecular markers for early diagnosis and prognosis of this syndrome. In addition, it is for crucial importance to identify the mutational profile associated to LS in Tunisian population allowing us to implement an oncogenetic counseling based on genetic tests specific to this population. This will help in early detection of individuals and families at high risk of developing LS and will consequently reduce mortality and morbidity due to the disease. Indeed LS guidelines outline specific surveillance and monitoring protocols based on MMR genes mutation testing and MMR proteins expression profile [5, 6]. The MMR system is composed of four proteins working in pairs (dimers-MLH1/PMS2 and MSH2/MSH6). These dimmers migrate to the nucleus to bind to the DNA. The formation of the complex is crucial for the stability, migration and the function of the complex [7].

LS is characterized by point mutations and/or large rearrangements in DNA MMR genes [8] resulting in a loss of MMR complex function and microsatellite instability (MSI) [9, 10]. The high penetrance mutations confer a predisposition to CRC in hereditary syndromes, responsible for about 50–80% of risk to develop CRC [11, 12]. However, there is a large variability in LS penetrance that is essentially dependent on low penetrance mutations and environmental factors. In fact, recently, germline mutations in DNA-repair genes (DRGs) have been reported in sporadic CRC, but their contribution to CRC risk and susceptibility is still unclear. Germline mutations in DRGs previously known to be linked to other inherited diseases could be involved in familial CRC predisposition [13]. Moreover, both germline and somatic variants in the exonuclease domains of DNA polymerase *ɛ* (*POLE*) and polymerase ${\bar{\text{d}}}$ (*POLD1*) have been reported to affect proofreading function and lead to an ultramutated phenotype [14]. Germline *POLE* variants can result in a LS phenotype and microsatellite instable CRCs. The exact effect of germline *POLE*/*POLD1* variants remains however, unclear [14–17].

The identification of an inherited mutation plays a crucial role in identifying at risk individuals and families for LS that are proposed for oncogenetic counseling [18]. However, Neither MMR mutated gene nor mutation type are associated to the onset age or the cancer type [3]. Thus, it is for crucial importance to search for other DRGs that could be implicated in the increase of CRC risk in patients with strong familial history. Moreover, underlying genotype–phenotype correlation in LS provides significant insights for oncogenetic counseling of familial CRC.

So far, very few clinical studies and genetic reports conducted on patients with LS in Tunisia have been published [19, 20]. Moussa et al. [20] have identified pathogenic mutations in MMR genes in only 11/31 LS suspected Tunisian families. Given limitations to this previous Tunisian study, the CCR susceptibility genes list could be expended with new DNA repair genes. In this study our main goal is to identify germline mutations associated to LS in a CRC Tunisian family with monozygotic twins and to assess factors associated with increasing cancer risk.

## Methods

### Patients

**Fig. 1:**
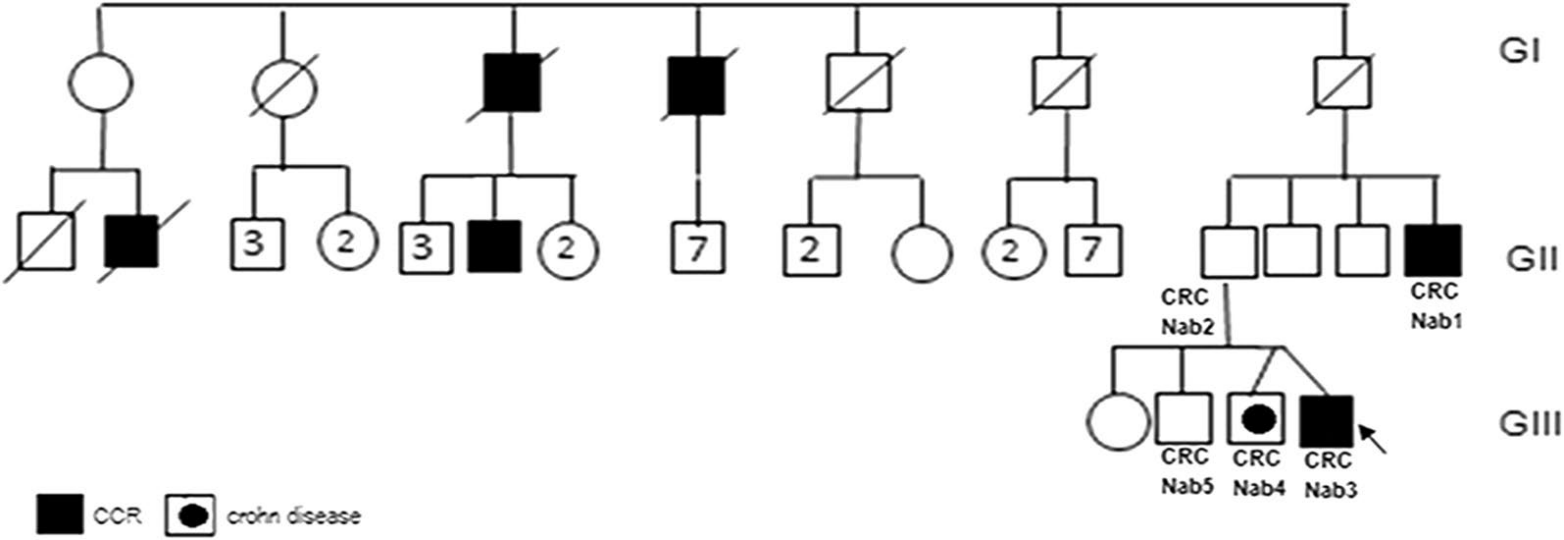
The familial pedigree of the HNPCC family. The index case family history included only colon cancer in second and third-degree relatives; it showed a tumor spectrum typical of LS I form

This study was conducted according to the declaration of Helsinki and to the approval of the Institutional reviewed board (IRB) of Institut Pasteur de Tunis. Five individuals, belonging to the same large Tunisian family, were investigated after written informed consent (Fig. 1). This family fulfill the Amsterdam I criteria for the diagnosis of Lynch syndrome. The index case was a man (CRCNab3), referred for a molecular diagnosis of LS to the Gastroenterology Department of Mohamed Tahar Maamouri Medical Hospital in Nabeul, Tunisia. He was diagnosed at 33-years-old with a well differentiated adenocarcinoma at the transverse colon (pT3 N1a of 6 cm × 6 cm × 2 cm) and treated with hemicolectomy. The index case has a monozygotic (MZ) twin diagnosed with crohn disease at the age of 35 (CRCNab4) and a brother who recently suffered from gastro-intestinal disconfort but considered as healthy (CRCNab5). Their father developed lymphoid hyperplasia at right colon without clinical significance (CRCNab2). Their paternal uncle (CRCNab1) was diagnosed with a sigmoidien well differentiated lieberkuhnien adenocarcinoma T3N0MX at 47-years-old treated with sigmoidectomy. The index case and his two brothers have smoking and/or alcohol consumption habits. The other investigated relatives have neither smoking nor alcohol consumption habits.

### Immunohistochemical study

To assess the expression of MMR proteins, we performed immunohistochemical labeling on Formalin Fixed Paraffin Embedded (FFPE) sample from the CRC of (CRCNab3), using the four primary antibodies against MMR system [anti-MLH1 (ES05), anti-MSH2 (25D12), anti-MSH6 (PU29) anti-PMS2 (M0R4G)] Leica Biosystems. We used a sporadic CRC with proficient MMR (pMMR) status as control.

### Targeted DNA repair genes panel conception (DRGs)

Library preparation for NGS was accomplished using the novel development of the HaloPlex assay that incorporates molecular barcodes for high-sensitivity sequencing as a custom design (HaloPlex^HS^). Using SureDesign (Agilent Technologies Inc.), probes were generated to cover the exons and 15 bp of the surrounding intronic sequences of a total of 87 candidate genes known to be involved in DNA repair disorders (the list of all analyzed genes is provided as Additional file 1). The size of the final target region was 251.689 kpb with 33828 amplicons and the mean coverage was 99.74% of the target region. Amplicon libraries were prepared, from genomic DNA of (CRCNab2) and (CRCNab3), using the HaloPlex^HS^ PCR target enrichment system dedicated to Ion Torrent PGM according to the manufacturer’s recommendations. Massively parallel sequencing was performed on an Ion Torrent PGM (Thermo Fisher Scientific). Raw data generated by the PGM sequencer were analysed using the in-house VarAft software version 2.5, which is freely available online (http://varaft.eu/index.php). We prioritized rare functional variants (missense, nonsense, splice site variants, and indels) and excluded variants with a Minor Allele Frequency (MAF) > 0.01 in dbSNP137, and 138, in the Exome Variant Server (http://evs.gs.washington.edu/EVS/), 1000 Genomes Project (http://www.1000genomes.org/), or Exome Aggregation Consortium database (ExAC), Cambridge, MA (http://exac.broadinstitute.org). A number of online tools were used to predict the functional impact and pathogenicity of the identified variants such as MutationTaster (http://www.mutationtaster.org/), PredictProtein (https://www.predictprotein.org/), PolyPhen (http://genetics.bwh.harvard.edu/pph2/), Combined Annotation Dependent Depletion (CADD) (http://cadd.gs.washington.edu/), SIFT (http://sift.bii.a-star.edu.sg/) and UMD predictor (http://umd-predictor.eu/). Variants not previously reported in healthy controls and classified as pathogenic were evaluated for sequencing depth and visually inspected using the Integrative Genomic Viewer (IGV) before validation by Sanger Sequencing.

### Sanger sequencing

PCR reactions were performed on genomic DNAs (gDNAs), following standard protocols, pursued by Sanger sequencing using an automated sequencer (ABI 3500; Applied Biosystems, Foster City, CA) using a cycle sequencing reaction kit (Big Dye Terminator kit, Applied Biosystems). Data were analyzed by BioEdit Sequence Alignment Editor Version 7.2.5. As the *POLE*/*POLD1* genes were not included in the HaloPlex gene panel list, the Sanger sequencing was used to screen for the following hotspot pathogenic mutations: p.L424V located in exon 13 of *POLE* gene, and p.S478N located in exon 11 of *POLD1* gene [15].

## Results

### Immunohistochemical pattern

**Fig. 2:**
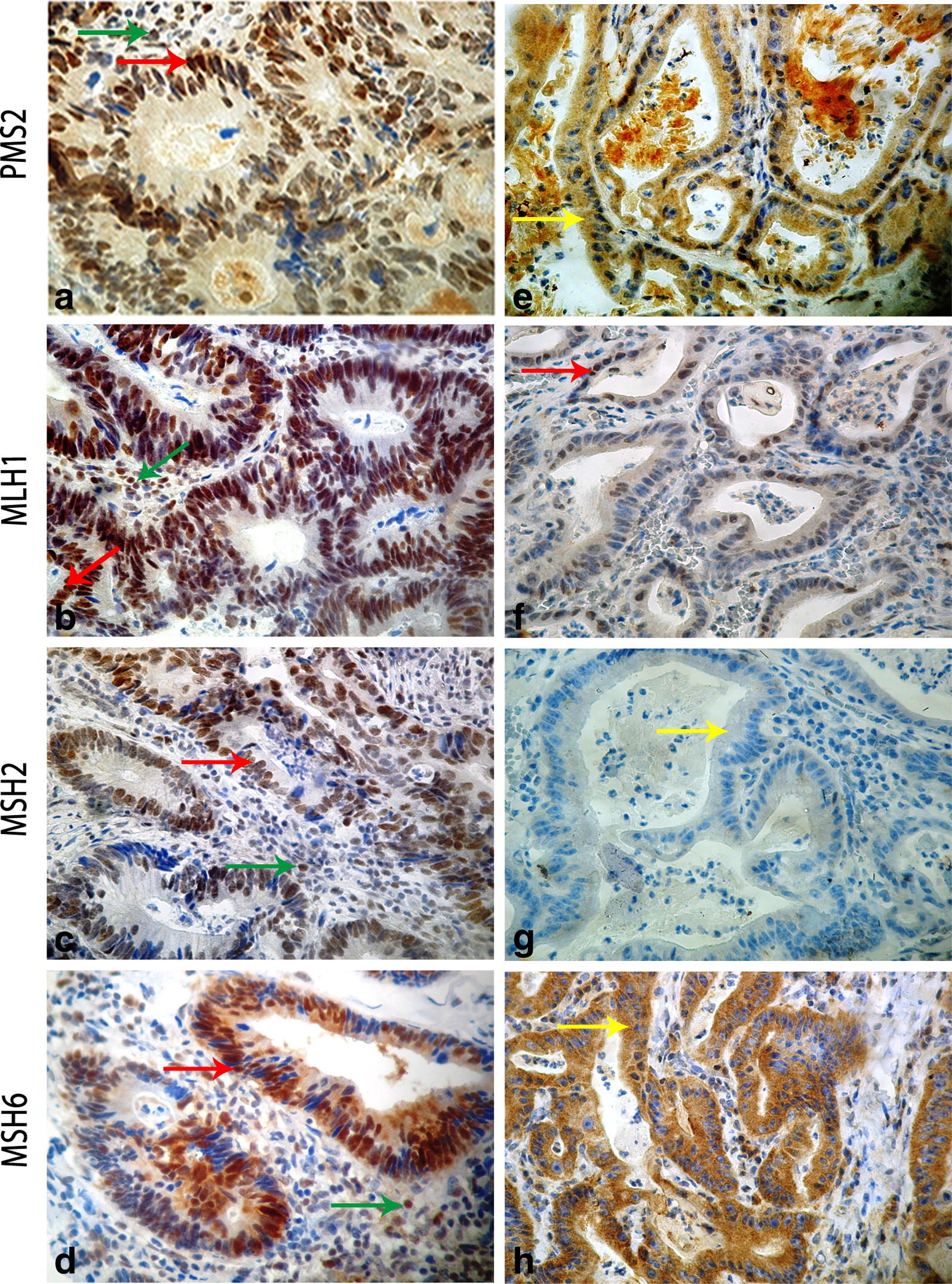
Representative MMR immunohistochemical expression patterns. Left panel: (**a**, **b**, **c** and **d**) are IHC pictures of a sporadic CRC sample with proficient MMR (pMMR). The pMMR sample staining shows strong nuclear expression in both tumor (red arrow) and adjacent stromal cells (internal control, green arrow) for the four used antibodies anti-PMS2 (**a**), anti-MLH1 (**b**), anti-MSH2 (**c**) and anti-MSH6 (**d**). Right panel: (**e**, **f**, **g** and **h**) are IHC pictures of the index case CRC with deficient MMR (dMMR). The dMMR proband staining showed: a loss of nuclear MSH2 expression in normal stromal and cancer cells (**g**) confirming the MMR deficiency, a nuclear incomplete staining for MLH1 (**f**) and a cytoplasmic staining for PMS2 (**e**) and MSH6 (**h**) (yellow arrow). Original magnifications (40x)

IHC result for (CRCNab3) showed an MSH2 nuclear expression loss in tumor and in stromal cells, a cytoplasmic staining for MSH6 and PMS2 and an incomplete nuclear staining for MLH1. The sporadic CRC sample with proficient MMR (pMMR), showed a positive nuclear staining in tumor cells as well as in adjacent normal cells (Fig. 2) with all the proteins.

### Pathogenic mutation detected by targeted DNA repair genes panel

Variants not previously reported in healthy controls and classified as pathogenic in ClinVar were evaluated for sequencing depth and visually inspected using the Integrative Genomic Viewer (IGV). After filtering strategies, described above, an *MSH2* mutation **(**c.1552C>T; p.Q518X) was detected in both, the index case (CRCNab3) and his father (CRCNab2) and it was selected as candidate for Sanger validation, among the other identified variations.

### Analysis of variants carried by the proband (CRCNab3) and absent in his father (CRCNab2)

**Fig. 3:**
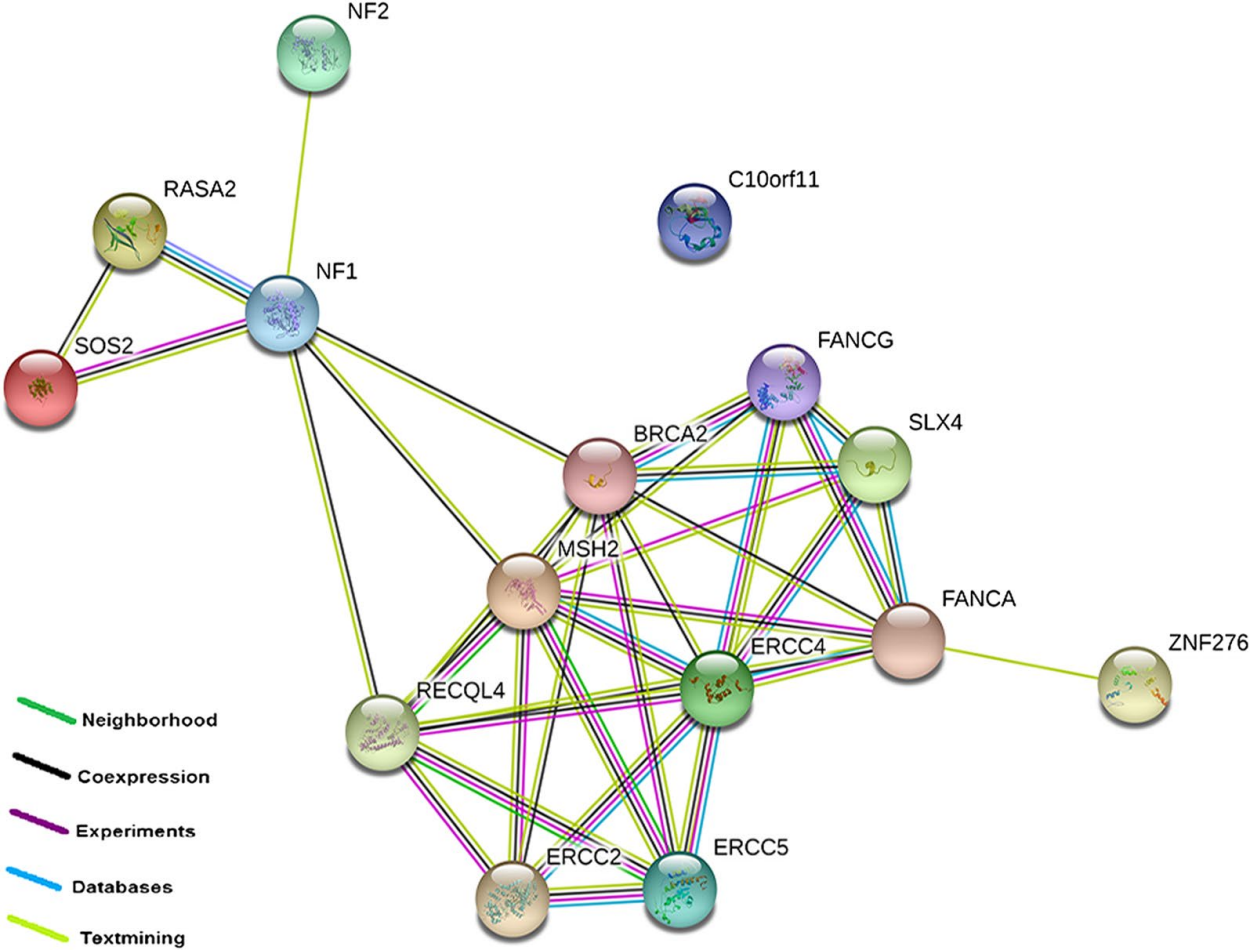
The protein–protein interaction network as analyzed by String software Version 11.0. The drawn edges represent the existence of different types of evidence used in predicting the associations

Eighty-seven variants on 60 genes have been detected in the proband (CRCNab3) not shared with his father (CRCNab2). After filtering steps (Table 1), fifteen non shared variants have been identified; 13 exonic variants, 1 splicing SNPs and 1 frameshift variant. We detected the following variations (Table 2): an SOS2 rare variant (rs532833599, MAF = 0.00019) and an LRMDA rare variant (rs763696041, MAF = 0.0005), *ERCC2* (exon5:c.360+3G>T), *ERCC4* (exon11: c.2422G>T: p.A808S), *ERCC5* (exon 1: c.8T>G: p.V3G), *BRCA2* exon 17 variation (c.7810C>A: p.L2604M), an exon 13 variation (c.2120G>T: p.C707F) in *RECQL4*, an exon 3 *SLX4* variation (c.742G>T: p.E248X) and two *NF1* variations in exon 25 (c.3259C>T: p.P1087S) and exon 43 (c.6623C>A:p. A2208D). Their respective protein interactions predictions are illustrated in Fig. 3 using String software Version 11.0.

**Table 1:** Variants filtering results of targeted DNA repair genes panel for the index case

Filtering conditions procedure	CRCNab3	
	Genes	Variants
Total number	87	268
Variations present for the proband case and absent in his father	60	87
Missense, nonsense, splice-site or frameshift variants	44	48
MAF < 0.1% in the 1000 genome, ExAC	35	37
CADD score ≥ 15	14	15

*MAF* minor allele frequency, *ExAC* the Exome Aggregation Consortium, *CADD* combined annotation dependent depletion

**Table 2:** Variants not shared between CRCNab3 and CRCNab2

Chr	Gene	NM Number	Func.refgene	Start	AA change	Frequency (exac/1000 g)	dbsnp	ClinVar	UMD prediction	SIFT_pred
10	LRMDA	NM_001305581	exon 6	78084234	c.592A>G:p.S198G	0.00005 (ExAC)	rs763696041	NA	Pathogenic	Tolerated
2	MSH2 (other then the confirmed one)	NM_000251	exon14	47705572	c.2372C>A:p.A791D	NA	NA	NA	Pathogenic	Tolerated
3	RASA2	NM_006506	exon 1	141206014	c.89A>T:p.D30V	NA	NA	NA	Pathogenic	Deleterious
8	RECQL4	NM_004260	exon 13	145739035	c.2120G>T:p.C707F	NA	NA	NA	NA	NA
16	FANCA; ZNF276	NM_000135	exon 43; UTR3	89805058	c.4319delA:p.Q1440fs	NA	NA	NA	NA	NA
9	FANCG	NM_004629	exon 14	35074189	c.1785C>A:p.S595R	NA	NA	NA	Polymorphism	Deleterious
14	SOS2	NM_006939	exon 18	50605450	c.2838A>T:p.L946F	0.000199681 (1000 g)	rs532833599	NA	Pathogenic	Deleterious
13	BRCA2	NM_000059	exon 17	32936664	c.7810C>A:p.L2604M	NA	NA	NA	Probably pathogenic	Deleterious
19	ERCC2	NM_000400	Splicing; intronic	45871885	exon5:c.360+3G>T	NA	NA	NA	NA	NA
16	ERCC4	NM_005236	exon 11	14041875	c.2422G>T:p.A808S	NA	NA	NA	Pathogenic	Tolerated
13	ERCC5	NM_000123	exon 1	103498624	c.8T>G:p.V3G	NA	NA	NA	Pathogenic	Deleterious
16	SLX4	NM_032444	exon 3	3656493	c.742G>T:p.E248X	NA	NA	NA	Pathogenic	NA
22	NF2	NM_000268	exon 5; intronic	30050687	c.489G>T:p.L163F	NA	NA	NA	Pathogenic	Deleterious
17	NF1	NM_001042492	exon 25	29559152	c.3259C>T:p.P1087S	NA	NA	NA	Pathogenic	Deleterious
			exon 43	29664581	c.6623C>A:p.A2208D	NA	NA	NA	Pathogenic	Deleterious

*ClinVar* clinical significance of the variation, *dbsnp* Single Nucleotide Polymorphism Database, *UMD predictor* a high-throughput sequencing compliant system for pathogenicity prediction, *SIFT* sorting intolerant from tolerant; predicting amino acid changes that affect protein function

### Sanger sequencing

**Fig. 4:**
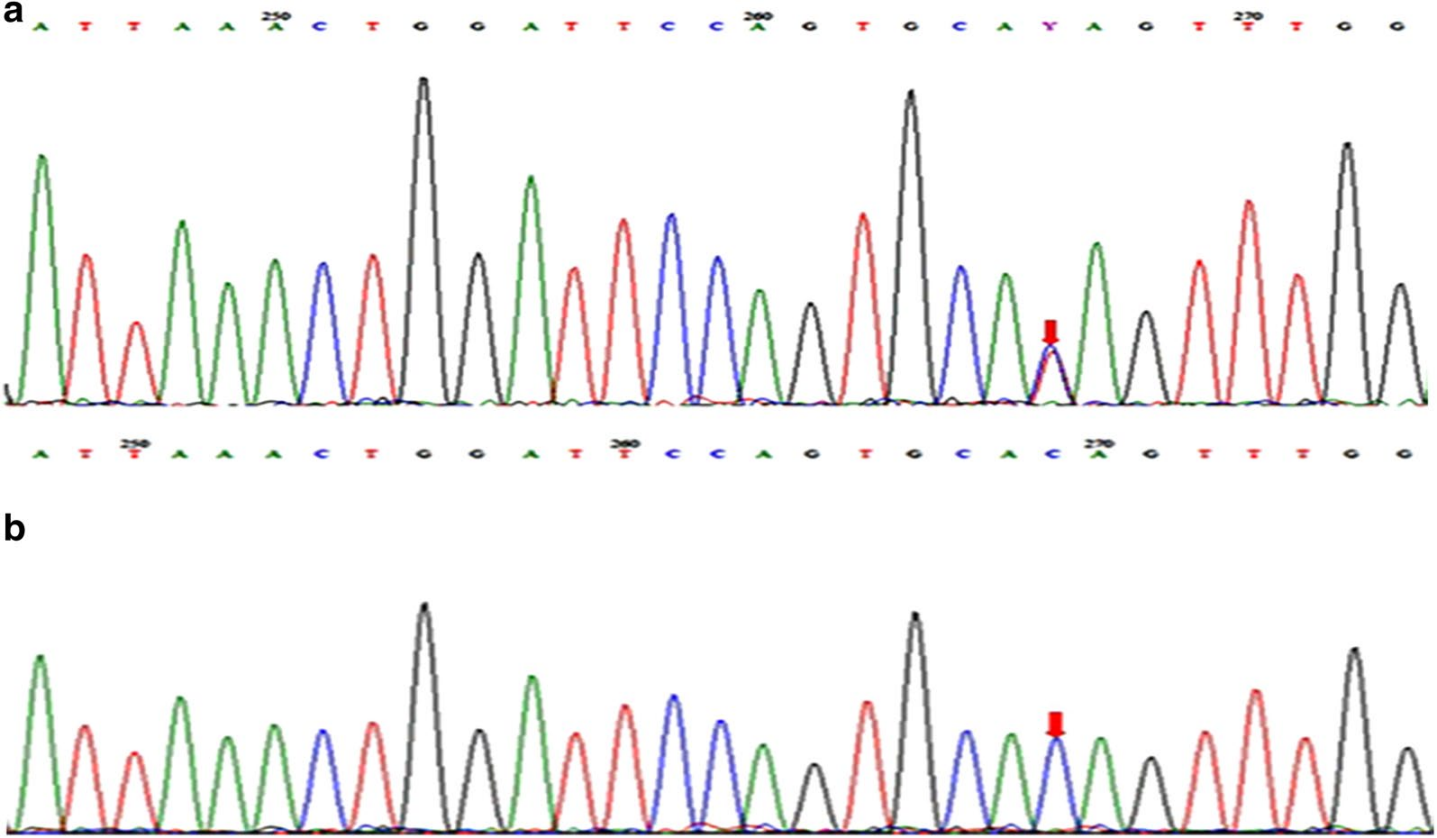
Sanger sequencing chromatograms of *MSH2* exon10 region: **a** control subject; **b** c.1552C>T in the index case (CRCNab3)

The mutation in the exon 10 of the *MSH2* gene (exon 10; c.1552C>T; p.Q518X) was confirmed by Sanger sequencing (Fig. 4). We first confirmed it on the index case (CRCNab3) and then in all investigated first and second degree relatives (CRCNab2, CRCNab1, CRCNab4 and CRCNab5). The consequence of this mutation was a stop gain variant (p.Q518X). The global and the local Minor Allele frequencies (MAF) of this variant are illustrated in Table 3. In addition, neither p.L424V in *POLE* nor p.S478N in *POLD1*, were found by Sanger sequencing in investigated members.

**Table 3:** Lifestyle habits, phenotypes and Sanger sequencing results of screened family members

Subject	Nab1	Nab2	Twins		Nab5	Global MAF	Local MAF	Localization	ClinVar	eQTL p-value for colon tissu
			Nab3	Nab4						
Age of onset	47	−	33	35	−					
Lifestyle habits (smoking and drinking)	Neither smoking nor alcohol intake	Neither smoking nor alcohol intake	+++ (consumption and exposure to smoking) +++ alcohol intake	++ smoking	+++ smoking +++ alcohol intake					
Phenotype	CRC	Unaffected	CRC	Crohn	Unaffected					
Sanger sequencing: MSH2 (rs63750780)	M	M	M	M	M	NA	0	Exonic	Pathogenic	NA

*MAF* minor allele frequency, *Global MAF* data from the 1000 genomes project, *Local MAF* from data available in our research laboratory of 59 whole exome sequencing of Tunisian subjects without CRC, *eQTLs* expression quantitative trait loci, *M* mutated (heterozygote), *NA* not available. Lifestyle habits of the investigated family members, including smoking and alcohol intake are summarized. *ClinVar* clinical significance of the variation

### In silico prediction of the (p.Q518X) detected mutation on the MSH2/MSH6 dimerization

**Fig. 5:**
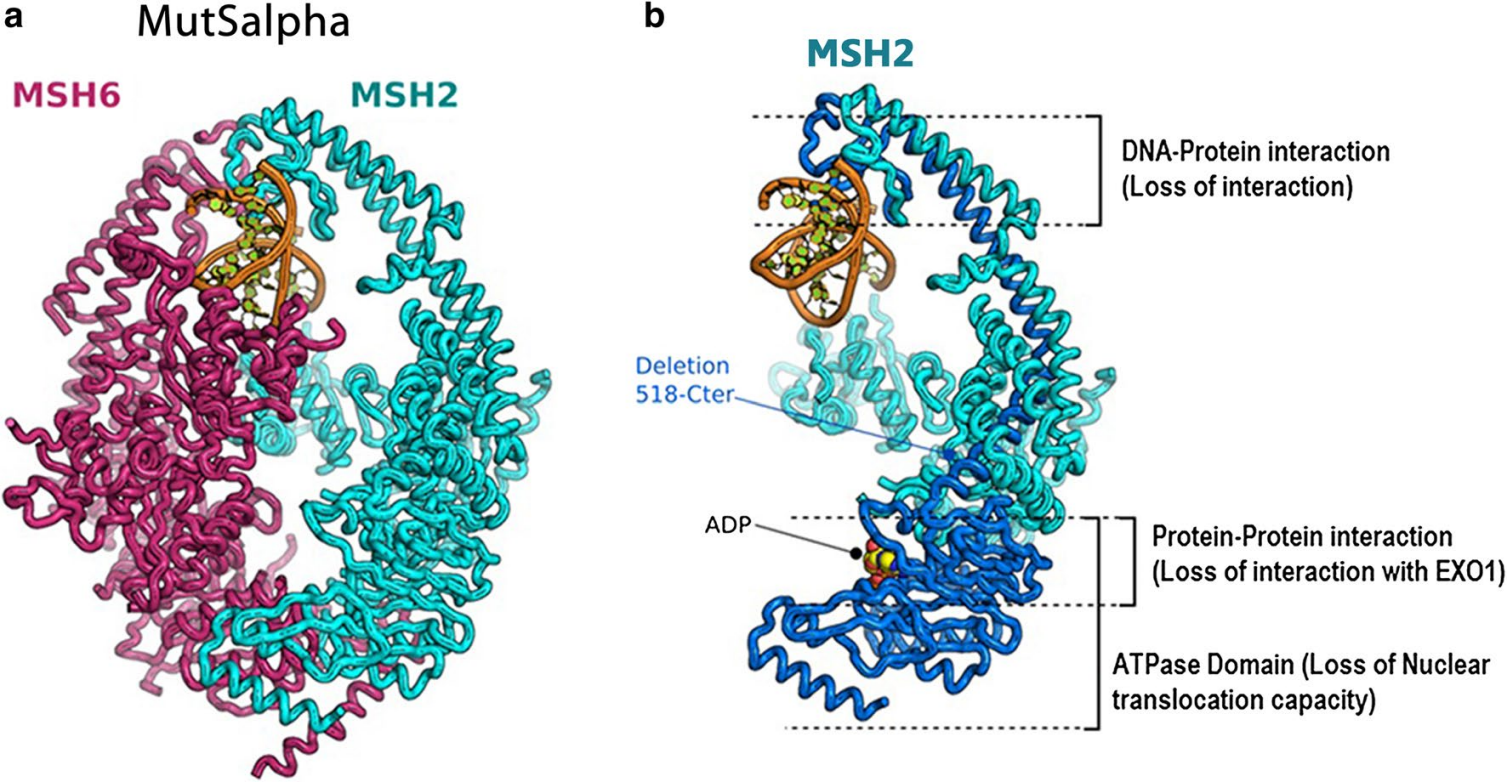
Mapping the likely outcomes of the mutation (p.Q518X) on the structure of the MutSalpha DNA lesion recognition complex. **a** The association between MSH6 and MSH2 proteins which dimeric form is capable of recognizing the damaged DNA is called MutSalpha. **b** Corresponds to the MSH2 protein in which we mapped the function segments downstream the stop codon insertion in dark blue. This results in loss of interaction between the two proteins (MSH2/MSH6) and a default of heterodimer formation (protein-protein interaction loss, DNA-protein interaction loss and nuclear translocation activity loss)

We have performed in silico prediction of the potential effect of this mutation on MSH2 protein structure and function. The Fig. 5 highlights the pathogenic effect of the identified *MSH2* mutation on MSH2/MSH6 heterodimerization. MutSalpha consists of the association of the MSH6 and MSH2 which dimeric form is capable of recognizing the damaged DNA (Fig. 5a). We mapped the function segments downstream the stop codon insertion in dark blue (Fig. 5b). This results in loss of interaction between different regions within the heterodimer (protein–protein interaction loss, DNA–protein interaction loss and nuclear translocation activity loss). The structure result was performed in the bases of the MSH2/MSH6 complex structure of Warren et al. [21] (PDB code: 2O8B). This finding is in perfect concordance with the IHC pattern showing an MSH6 cytoplasmic accumulation and a loss of MSH2 expression.

## Discussion

LS penetrance is highly variable and the reasons for this have not been fully elucidated. Peters et al. [22] affirmed that it remains critical that we stay on the path to uncover the complete genetic architecture of CRC to more fully understand the etiology of the disease. In Tunisian population, MMR germline mutations are responsible for at least 35.5% of CRC developed in patients with personal or familial history suggestive of Lynch syndrome [20]. In the only molecular Tunisian study by Moussa et al. [20], the entire coding regions, splice junctions and promoter regions of MLH1 and MSH2 were screened for the presence of point mutations. The following mutations in *MSH2* and *MLH1* were described in their investigated Tunisian LS families; *MSH2* (p.Gln402X, p.Pro472ThrfsX4, p.Arg243Gln, p.Ser281X and p.Gly713ArgfsX4) and *MLH1* (p.Ala111Asp, p.Gln197ArgfsX8, p.His718Tyr, p.Lys392SerfsX9, p.Arg226X and p.Glu153PhefsX8) [20]. MSH6 was analyzed but no mutation was founded. In this previous Tunisian study 64.5% (20/31) of families with suspicion of LS remain with undiscovered mutations in MMR genes. Thus, they suggest that other genes could predispose to non polyposis CRC. In this context, we described herein an interesting Tunisian family with strong history of colon cancer affecting three generations with a tumor spectrum specific to LS I form (Fig. 1). Genetic investigation using targeted sequencing DNA repair genes panel revealed, among detected variations, a single nucleotide substitution (c.1552C>T) in *MSH2* in the proband (CRCNab3) and in his father (CRC Nab2). It was confirmed by Sanger sequencing (Fig. 2) and identified in the 47-years-old paternal uncle, diagnosed with a sigmoidien CRC and in three of the proband first degree relatives previously cited. This mutation is identified for the first time in a Tunisian LS family and was reported once by Fidalgo et al. [23] in an index case of LS Portuguese family. Fidalgo et al. [23] have confirmed the pathogenicity of this mutation by various approaches such as protein truncation test (PTT), single strand conformation polymorphism (SSCP), heteroduplex analysis (HA) and denaturing gradient gel electrophoresis (DGGE) as well [23]. The effect of this mutation is a premature stop codon (p.Q518X) which is already reported in InSiGHT variant databases (https://www.insight-group.org/variants/databases/) as a pathogenetic variant. The distribution of such rare mutation could be explained either by the only achieved molecular LS Tunisian study or by the scarcity of this mutation all over the world. The In silico prediction of the effect of this mutation on (MSH2·MSH6 heterodimer), crucial for MMR complex function [21, 24], revealed that its pathogenicity affects allosteric interactions between different regions within the heterodimer; loss of MSH2 ATPase Domain (loss of nuclear translocation capacity), loss of interaction with EXO1 and Loss of DNA–protein interaction. This will be translated in IHC expression profile by the loss of MSH2 nuclear expression and a cytoplasmic MSH6 accumulation. In almost all published articles using IHC analysis, the MSI phenotype is assigned following the loss of expression of MLH1 or MSH2 [20, 25, 26]. CRCNab3 phenotype was consistent with deficient MMR system (dMMR) linked to LS. The In situ functional effect of this mutation (c.1552C>T, p.Q518X) was confirmed by the obtained immunohistochemical pattern. Our IHC results are in concordance with molecular ones, supporting the evidence that MMR protein loss is explained notably by the pathogenic mutation in corresponding MMR gene. Moreover, IHC interpretation guidelines for cytoplasmic MMR staining bears no exact significance [27–29]. There are no data as yet to indicate that its presence is reflective of protein deficiency. Our results bring evidence that cytoplasmic staining could be taken into account to the evaluation of function loss within MSH2/MSH6 heterodimer. To the best of our knowledge, this is the first Tunisian study describing the effect of (c.1552C>T; p.Q518X) *MSH2* mutation on MSH2/MSH6 complex heterodimerization, confirmed by IHC. Furthermore, it has been reported that in MSI cases, the presence of the *BRAF*^*V600E*^ hotspot mutation excludes the diagnosis of LS, and the clinical utility of the combination of MMR and BRAF status is well established [30]. In this context, no somatic *BRAF*^*V600E*^ mutation was detected in our investigated index case, confirming the LS.

In this work, the investigated family members share the same *MSH2* pathogenic mutation (c.1552C>T, p.Q518X) with different phenotypes, suggesting hence, an important role of microenvironment and/or other DRGs mutations. The two CRC cases (CRCNab1 and CRCNab3) had both the *MSH2* pathogenic mutation. CRCNab3 has alcohol and smoking habits that CRCNab1 has not. These two CRC patients showed some differences in colon tumor localization and age of disease onset. The 65 years-old unaffected mutation carrier father (CRC Nab2) had a healthy life style contrary to his 34 years-old unaffected mutation carrier son (CRC Nab5) who is a smoker and alcohol consumer.

Interestingly, MZ twins provide a model to investigate environmental effects on disease development and progression [31, 32]. The proband MZ twin (CRC Nab4) carried the *MSH2* pathogenic mutation (c.1552C>T, p.Q518X) without alcohol habits. He has developed crohn disease (CD) at 35 years-old. It was already known that chronic inflammation creates a microenvironment suitable for the disease progression [33]. dosSantos [34], has identified that a pro-inflammatory state is the cornerstone in the association between CD and CRC, justifying the fact that CD might be a risk factor for CRC. She added that a family history of CRC is an important factor that doubles the risk of CRC in patients with CD. In our study, discordant twins’ habits allow us to suggest that they could directly or indirectly affect DNA changes independently of their mutational status. Alcohol consumption and cigarette smoking are considered as major risk factors for gastrointestinal cancer, including colorectal cancer [35]. The World Cancer Research Fund and the American Institute of Cancer Research suggests that excessive alcohol consumption enhance the risk of colorectal cancer. As a result of cumulative evidence from epidemiological studies, colorectal cancer has been listed as a pathology linked to alcohol intake and cigarette smoking [36]. This MZ twins discordance, pointed the important roles of environmental and modifiable factors in relation to gene–environment interactions in the prevention of CRC [37]. Studies of gene–environment interactions in families are crucial in providing potential insights for developing prevention strategies against CRC [38, 39]. Public health policies to prevent this cancer should include modification of alcohol intake habits, especially among individuals at increased risk [35, 40].

Carcinogenesis model in sporadic and hereditary CRC are based on the accumulation of mutations which is the critical determinant of tumorigenesis [41]. Currently, through genome-wide association studies, it has become possible to evaluate the role of common low-penetrance genetic modifiers and how they can affect disease expression that occurs both within families or individuals with similar MMR gene status [22]. Donald et al. [42], have conducted a meta-analysis to evaluate the role and effects of common low-penetrance genetic polymorphisms for a better understanding of their association with CRC risk in individuals belonging to LS families. They failed to uncover consistent evidence that LS phenotype is influenced by the effects of low penetrance modifiers. Weigl et al. [43] have identified that both family history and the identified genetic variants carry essential risk information and their combination provide great potential for CRC risk stratification. In this context, besides the pathogenic *MSH2* mutation (c.1552C>T; p.Q518X) shared by the index case and his father, the Table 2 summarize other pinpointed pathogenic variants present on the index case not shared with his father.

It is widely recognized that environmental carcinogens induce DNA damage, which could in turn induce genomic instability [44]. The bulky DNA adducts generated by tobacco carcinogens are mainly repaired by nucleotide excision repair (NER). NER is the most common pathway for repairing bulky DNA lesions and maintaining genomic stability. Different key proteins are involved in this process including; ERCC2 (XPD) which accomplish 3′–5′ unwinding of the DNA strands of the damaged site, while the damaged DNA is excised at 5′ site by XPF (ERCC4)-ERCC1 heterodimer and at 3′ site by ERCC5 (XPG), which is an MSH2 and RECQL4 neighbor (Fig. 5). Aberrant expression of key NER factors alters NER capacity, thus threatening genomic stability and integrity [45]. In our study, we noted the following variants: *ERCC2* (exon5:c.360+3G>T), *ERCC4* (exon 11: c.2422G>T: p.A808S) and *ERCC5* (exon 1: c.8T>G: p.V3G). Therefore, our identified alterations in the index case not shared with his father in NER pathway members could alter the efficacy of DNA repair and might enhance colorectal cancer risk. Only few studies have examined the contribution of SNPs in NER pathway genes to CRC risk [46, 47]. Our study is the first Tunisian one which highlights that variants in some members of the xeroderma pigmentosum (XP) genes family could play an important role in colorectal cancer increased risk.

Other cellular DNA repair pathways, such as base excision repair (BER), double-strand break repair (DSBR), and homologous recombination repair (HRR) also play important roles in the carcinogenesis process by repairing single strand and double strand DNA breaks induced by smoking, ionizing radiation, and other DNA damaging agents [47]. *BRCA2* is a member of the HRR pathway, which restores the integrity of double-strand DNA breaks [48]. Inherited mutations in HRR genes have long been known to increase the risk of several cancers, including breast, ovarian, prostate and pancreatic cancers [49]. Risch et al. [50] reported that there is an increased risk for colon cancer in *BRCA2* families. We identified a *BRCA2* exon 17 variation (c.7810C>A: p.L2604M) in the index case.

*BRCA2* is co-expressed with RecQ protein-like 4 (*RECQL4*) which is a key member of the RecQ family and plays an important role in the initiation of DNA replication, progression of stalled replication forks, and telomere maintenance, as well as in the repair of DNA DSB via the HRR pathway [51]. Mutations of the *RECQL4* gene are associated with the rare type II Rothmund–Thomson syndrome, which has a propensity for osteosarcomas [52]. However, recent studies have shown that *RECQL4* acts as a tumor-promotor in some cancers, such as prostate cancer, colorectal cancer, and breast cancer [52–55]. We detected (c.2120G>T: p.C707F) variation in the exon 13 of *RECQL4*. This is the first description of this variation in patients with CRC. Structure-specific endonuclease subunit (SLX4) encodes a Fanconi anemia-related protein that is required for repair of specific types of DNA lesions and critical for cellular responses to replication fork failure. Lee et al. [56] suggest that frameshift mutations of *SLX4* may play a cancer-related role in limited cases of CRCs. We found an exon 3 *SLX4* variation (c.742G>T: p.E248X).

*NF1* which plays a role as a tumor suppressor gene [57] is co-expressed with *RECQL4* and *BRCA2*. To date, the association between NF1 and adenocarcinoma of the gastrointestinal tract is thought to be casual. However, Li et al. [58] suggested that germline mutations in *NF1* can occur in somatic cells and contribute to cancer development. Indeed, Seminog and Goldacre [59] observed that *NF1* patients were at high risk of colon and recto-sigmoid junction cancer when compared with the general population. We detected *NF1* variations in exon 25 (c.3259C>T: p.P1087S) and exon 43 (c.6623C>A:p. A2208D).

These actionable genes are not part of the recommended germline testing for individuals with familial CRC. The Fig. 3 showed their respectively protein–protein interactions, supporting the hypothesis that other variants unusually described in CRC might explain in part the phenotypic difference between the father and his son (CRCNab3). Thus, patients with multiple low penetrance SNPs could be experiencing an additive effect to increase CRC risk through gene–gene interactions. Confirmation of these identified variants by Sanger sequencing could be of important output regarding LS genetic profiling.

## Conclusion

In overall, a better understanding of the genotype–phenotype correlation associated to LS may lead to implement a personalized oncogenetic counseling of individuals with particular mutational genetic profiles in terms of their risk management. Since, promoting a universal LS screening was the project aim of the International Mismatch Repair Consortium (IMRC), our study results are taking part from the conducted research projects in this field. Therefore, further studies are needed, with more particular attention to low penetrance modifier variants in order to better define the genotype–phenotype correlation and risk evaluation of colorectal carcinoma in LS context. Further conclusions regarding CRC-risk events should be based on a larger series of patients and families.

## Additional file

**Additional file 1.** Targeted DNA repair genes panel list (87 genes).

## Data Availability

All data generated or analyzed during this study are included in this published article and its Additional file.

## Abbreviations

DNA: deoxyribonucleic acid
gDNA: genomic DNA
MAF: minor allele frequency
NGS: next generation sequencing
PCR: polymerase chain reaction
SIFT: sorting intolerant from tolerant
SNP: single nucleotide polymorphism
VarAFT: variant annotation and filtering tool
IGV: integrative genomic viewer
MMR: mismatch repair
BER: base excision repair
DSBR: double-strand break repair
HRR: homologous recombination repair
NER: nucleotide excision repair
XP: xeroderma pigmentosum
IMRC: International Mismatch Repair Consortium
CD: crohn disease
MZ: monozygotic
FFPE: formalin fixed paraffin embedded
IHC: immunohistochemistry
CRC: colorectal cancer
LS: Lynch syndrome
DRGs: DNA-repair genes
MSI: microsatellite instability

## References

[1] Mauri G, Sartore-Bianchi A, Russo AG, Marsoni S, Bardelli A, Siena S (2019). Early-onset colorectal cancer in young individuals. Mol Oncol.

[2] Lorans M, Dow E, Macrae FA, Winship IM, Buchanan DD (2018). Update on hereditary colorectal cancer: improving the clinical utility of multigene panel testing. Clin Colorectal Cancer.

[3] Liccardo R, De Rosa M, Duraturo F (2018). Same MSH2 gene mutation but variable phenotypes in 2 families with lynch syndrome: two case reports and review of genotype-phenotype correlation. Clin Med Insights Case Rep.

[4] Lv XP (2017). Gastrointestinal tract cancers: genetics, heritability and germ line mutations. Oncol Lett.

[5] Mishra N, Hall J (2012). Identification of patients at risk for hereditary colorectal cancer. Clin Colon Rectal Surg.

[6] Richards S, Aziz N, Bale S, Bick D, Das S, Gastier-Foster J (2015). Standards and guidelines for the interpretation of sequence variants: a joint consensus recommendation of the American College of Medical Genetics and Genomics and the Association for Molecular Pathology. Genet Med.

[7] Ismael NE, El Sheikh SA, Talaat SM, Salem EM (2017). Mismatch repair proteins and microsatellite instability in colorectal carcinoma (MLH1, MSH2, MSH6 and PMS2): histopathological and immunohistochemical study. Open Access Maced J Med Sci.

[8] Duraturo F, Cavallo A, Liccardo R, Cudia B, De Rosa M, Diana G (2013). Contribution of large genomic rearrangements in Italian Lynch syndrome patients: characterization of a novel alu-mediated deletion. Biomed Res Int.

[9] Romero A, Garre P, Valentin O, Sanz J, Perez-Segura P, Llovet P (2013). Frequency and variability of genomic rearrangements on MSH2 in Spanish Lynch syndrome families. PLoS ONE.

[10] Zhang L (2008). Immunohistochemistry versus microsatellite instability testing for screening colorectal cancer patients at risk for hereditary nonpolyposis colorectal cancer syndrome. Part II. The utility of microsatellite instability testing. J Mol Diagn.

[11] Cooper DN, Krawczak M, Polychronakos C, Tyler-Smith C, Kehrer-Sawatzki H (2013). Where genotype is not predictive of phenotype: towards an understanding of the molecular basis of reduced penetrance in human inherited disease. Hum Genet.

[12] Liccardo R, De Rosa M, Izzo P, Duraturo F (2017). Novel implications in molecular diagnosis of Lynch syndrome. Gastroenterol Res Pract.

[13] AlDubayan SH, Giannakis M, Moore ND, Han GC, Reardon B, Hamada T (2018). Inherited DNA-repair defects in colorectal cancer. Am J Hum Genet.

[14] Castellucci E, He T, Goldstein DY, Halmos B, Chuy J (2017). DNA polymerase varepsilon deficiency leading to an ultramutator phenotype: a novel clinically relevant entity. Oncologist.

[15] Esteban-Jurado C, Gimenez-Zaragoza D, Munoz J, Franch-Exposito S, Alvarez-Barona M, Ocana T (2017). POLE and POLD1 screening in 155 patients with multiple polyps and early-onset colorectal cancer. Oncotarget.

[16] Jansen AM, van Wezel T, van den Akker BE, Ventayol Garcia M, Ruano D, Tops CM (2016). Combined mismatch repair and POLE/POLD1 defects explain unresolved suspected Lynch syndrome cancers. Eur J Hum Genet.

[17] Elsayed FA, Kets CM, Ruano D, van den Akker B, Mensenkamp AR, Schrumpf M (2015). Germline variants in POLE are associated with early onset mismatch repair deficient colorectal cancer. Eur J Hum Genet.

[18] Cohen SA, Leininger A (2014). The genetic basis of Lynch syndrome and its implications for clinical practice and risk management. Appl Clin Genet.

[19] Amira AT, Mouna T, Ahlem B, Raoudha A, Majid BH, Amel H (2014). Immunohistochemical expression pattern of MMR protein can specifically identify patients with colorectal cancer microsatellite instability. Tumour Biol.

[20] Moussa SA, Moussa A, Kourda N, Mezlini A, Abdelli N, Zerimech F (2011). Lynch syndrome in Tunisia: first description of clinical features and germline mutations. Int J Colorectal Dis.

[21] Warren JJ, Pohlhaus TJ, Changela A, Iyer RR, Modrich PL, Beese LS (2007). Structure of the human MutSalpha DNA lesion recognition complex. Mol Cell.

[22] Peters U, Bien S, Zubair N (2015). Genetic architecture of colorectal cancer. Gut.

[23] Fidalgo P, Almeida MR, West S, Gaspar C, Maia L, Wijnen J (2000). Detection of mutations in mismatch repair genes in Portuguese families with hereditary non-polyposis colorectal cancer (HNPCC) by a multi-method approach. Eur J Hum Genet.

[24] Reyes GX, Schmidt TT, Kolodner RD, Hombauer H (2015). New insights into the mechanism of DNA mismatch repair. Chromosoma.

[25] Mensenkamp AR, Vogelaar IP, van Zelst-Stams WA, Goossens M, Ouchene H, Hendriks-Cornelissen SJ (2014). Somatic mutations in MLH1 and MSH2 are a frequent cause of mismatch-repair deficiency in Lynch syndrome-like tumors. Gastroenterology.

[26] Gray PN, Tsai P, Chen D, Wu S, Hoo J, Mu W (2018). TumorNext-Lynch-MMR: a comprehensive next generation sequencing assay for the detection of germline and somatic mutations in genes associated with mismatch repair deficiency and Lynch syndrome. Oncotarget.

[27] Remo A, Fassan M, Lanza G, on behalf of AIFEG and GIPAD (2015). Immunohistochemical evaluation of mismatch repair proteins in colorectal carcinoma: the AIFEG/GIPAD proposal. Pathologica.

[28] McCarthy AJ, Capo-Chichi JM, Spence T, Grenier S, Stockley T, Kamel-Reid S (2019). Heterogenous loss of mismatch repair (MMR) protein expression: a challenge for immunohistochemical interpretation and microsatellite instability (MSI) evaluation. J Pathol Clin Res.

[29] Jung J, Kang Y, Lee YJ, Kim E, Ahn B, Lee E (2017). Comparison of the mismatch repair system between primary and metastatic colorectal cancers using immunohistochemistry. J Pathol Transl Med.

[30] Funkhouser WK, Lubin IM, Monzon FA, Zehnbauer BA, Evans JP, Ogino S (2012). Relevance, pathogenesis, and testing algorithm for mismatch repair-defective colorectal carcinomas: a report of the association for molecular pathology. J Mol Diagn.

[31] Allione A, Marcon F, Fiorito G, Guarrera S, Siniscalchi E, Zijno A (2015). Novel epigenetic changes unveiled by monozygotic twins discordant for smoking habits. PLoS ONE.

[32] Marcon F, Carotti D, Andreoli C, Siniscalchi E, Leopardi P, Caiola S (2013). DNA damage response in monozygotic twins discordant for smoking habits. Mutagenesis.

[33] Freeman HJ (2008). Colorectal cancer risk in Crohn’s disease. World J Gastroenterol.

[34] dosSantos SCD, Barbosa LER (2017). Crohn's disease: risk factor for colorectal cancer. J Coloproctol..

[35] Hughes LAE, Simons C, van den Brandt PA, van Engeland M, Weijenberg MP (2017). Lifestyle, diet, and colorectal cancer risk according to (Epi)genetic instability: current evidence and future directions of molecular pathological epidemiology. Curr Colorectal Cancer Rep.

[36] Cho S, Shin A, Park SK, Shin HR, Chang SH, Yoo KY (2015). Alcohol drinking, cigarette smoking and risk of colorectal cancer in the korean multi-center cancer cohort. J Cancer Prev.

[37] Shiao SPK, Grayson J, Yu CH, Wasek B, Bottiglieri T (2018). Gene environment interactions and predictors of colorectal cancer in family-based, multi-ethnic groups. J Personal Med.

[38] Campbell PT, Curtin K, Ulrich CM, Samowitz WS, Bigler J, Velicer CM (2009). Mismatch repair polymorphisms and risk of colon cancer, tumour microsatellite instability and interactions with lifestyle factors. Gut.

[39] Visser A, Vrieling A, Murugesu L, Hoogerbrugge N, Kampman E, Hoedjes M (2017). Determinants of adherence to recommendations for cancer prevention among Lynch syndrome mutation carriers: a qualitative exploration. PLoS ONE.

[40] Vanella G, Archibugi L, Stigliano S, Capurso G (2018). Alcohol and gastrointestinal cancers. Curr Opin Gastroenterol.

[41] Fearon ERVB (1990). A genetic model for colorectal tumorigenesis. Cell.

[42] Donald N, Malik S, McGuire JL, Monahan KJ (2018). The association of low penetrance genetic risk modifiers with colorectal cancer in lynch syndrome patients: a systematic review and meta-analysis. Fam Cancer.

[43] Weigl K, Chang-Claude J, Knebel P, Hsu L, Hoffmeister M, Brenner H (2018). Strongly enhanced colorectal cancer risk stratification by combining family history and genetic risk score. Clin Epidemiol.

[44] Basu AK (2018). DNA damage, mutagenesis and cancer. Int J Mol Sci.

[45] Liu J, Li H, Sun L, Feng X, Wang Z, Yuan Y (2018). The differential expression of core genes in nucleotide excision repair pathway indicates colorectal carcinogenesis and prognosis. Biomed Res Int.

[46] Mucha B, Pytel D, Markiewicz L, Cuchra M, Szymczak I, Przybylowska-Sygut K (2018). Nucleotide excision repair capacity and XPC and XPD gene polymorphism modulate colorectal cancer risk. Clin Colorectal Cancer.

[47] Aggarwal N, Donald ND, Malik S, Selvendran SS, McPhail MJ, Monahan KJ (2017). The association of low-penetrance variants in DNA repair genes with colorectal cancer: a systematic review and meta-analysis. Clin Transl Gastroenterol.

[48] Mladenov E, Magin S, Soni A, Iliakis G (2016). DNA double-strand-break repair in higher eukaryotes and its role in genomic instability and cancer: cell cycle and proliferation-dependent regulation. Semin Cancer Biol.

[49] Iqbal J, Ragone A, Lubinski J, Lynch HT, Moller P, Ghadirian P (2012). The incidence of pancreatic cancer in BRCA1 and BRCA2 mutation carriers. Br J Cancer.

[50] Risch HA, McLaughlin JR, Cole DE, Rosen B, Bradley L, Kwan E (2001). Prevalence and penetrance of germline BRCA1 and BRCA2 mutations in a population series of 649 women with ovarian cancer. Am J Hum Genet.

[51] Singh DK, Popuri V, Kulikowicz T, Shevelev I, Ghosh AK, Ramamoorthy M (2012). The human RecQ helicases BLM and RECQL4 cooperate to preserve genome stability. Nucleic Acids Res.

[52] Yin J, Kwon YT, Varshavsky A, Wang W (2004). RECQL4, mutated in the Rothmund–Thomson and RAPADILINO syndromes, interacts with ubiquitin ligases UBR1 and UBR2 of the N-end rule pathway. Hum Mol Genet.

[53] Su Y, Meador JA, Calaf GM, De-Santis LP, Zhao Y, Bohr VA (2010). Human RecQL4 helicase plays critical roles in prostate carcinogenesis. Can Res.

[54] Lao VV, Welcsh P, Luo Y, Carter KT, Dzieciatkowski S, Dintzis S (2013). Altered RECQ helicase expression in sporadic primary colorectal cancers. Transl Oncol.

[55] Fang H, Nie L, Chi Z, Liu J, Guo D, Lu X (2013). RecQL4 helicase amplification is involved in human breast tumorigenesis. PLoS ONE.

[56] Lee JH, An CH, Kim MS, Yoo NJ, Lee SH (2018). Rare frameshift mutations of putative tumor suppressor genes CSMD1 and SLX4 in colorectal cancers. Pathol Res Pract.

[57] Kim IY, Cho MY, Kim YW (2014). Synchronous multiple colonic adenocarcinomas arising in patient with neurofibromatosis type 1. Ann Surg Treat Res.

[58] Li Y, Bollag G, Clark R, Stevens J, Conroy L, Fults D, Ward K, Friedman E, Samowitz W, Robertson M (1992). Somatic mutations in the neurofibromatosis 1 gene in human tumors. Cell.

[59] Seminog OO, Goldacre MJ (2013). Risk of benign tumours of nervous system, and of malignant neoplasms, in people with neurofibromatosis: population-based record-linkage study. Br J Cancer.

